# Neural-Network-Based Approaches for Optimization of Machining Parameters Using Small Dataset

**DOI:** 10.3390/ma15030700

**Published:** 2022-01-18

**Authors:** Aleksandar Kosarac, Cvijetin Mladjenovic, Milan Zeljkovic, Slobodan Tabakovic, Milos Knezev

**Affiliations:** 1 Faculty of Mechanical Engineering, University of East Sarajevo, 71123 Istočno Sarajevo, Bosnia and Herzegovina; aleksandar.kosarac@ues.rs.ba; 2 Department of Production Engineering, Faculty of Technical Sciences, University of Novi Sad, 21000 Novi Sad, Serbia; milanz@uns.ac.rs (M.Z.); tabak@uns.ac.rs (S.T.); knezev@uns.ac.rs (M.K.)

**Keywords:** artificial neural networks, surface roughness, design of experiment, small dataset

## Abstract

Surface quality is one of the most important indicators of the quality of machined parts. The analytical method of defining the arithmetic mean roughness is not applied in practice due to its complexity and empirical models are applied only for certain values of machining parameters. This paper presents the design and development of artificial neural networks (ANNs) for the prediction of the arithmetic mean roughness, which is one of the most common surface roughness parameters. The dataset used for ANN development were obtained experimentally by machining AA7075 aluminum alloy under various machining conditions. With four factors, each having three levels, the full factorial design considers a total of 81 experiments that have to be carried out. Using input factor-level settings and adopting the Taguchi method, the experiments were reduced from 81 runs to 27 runs through an orthogonal design. In this study we aimed to check how reliable the results of artificial neural networks were when obtained based on a small input-output dataset, as in the case of applying the Taguchi methodology of planning a four-factor and three-level experiment, in which 27 trials were conducted. Furthermore, this paper considers the optimization of machining parameters for minimizing surface roughness in machining AA7075 aluminum alloy. The results show that ANNs can be successfully trained with small data and used to predict the arithmetic mean roughness. The best results were achieved by backpropagation multilayer feedforward neural networks using the BR algorithm for training.

## 1. Introduction

Surface quality is one of the most important indicators of the quality of machined parts [1]. The arithmetic mean roughness (Ra) represents a measure of the surface quality [2]. The arithmetic mean roughness is influenced by machining parameters and tool geometry. The analytical method of defining the arithmetic mean roughness is not applied in practice due to its complexity and empirical models are applied only for certain values of machining parameters.

The design of machine parts very often focuses on dimensional and form tolerances. In cases where the quality of the surface has significant importance and requires an indicator, the arithmetic mean roughness (Ra) is often used. Some researchers have investigated the influence of cutting parameters (cutting speed, feed rate, axial and radial depth of the cut) on the arithmetic mean roughness (Ra) [3,4,5,6,7,8,9].

It can be noted that cutting speed, feed rate and the depth of the cut are the most dominant factors in these studies, even though some researchers have used other factors which can influence surface roughness, such as vibration or tool wear [10,11,12].

Some of the researchers have examined the influence of different cooling/lubricating techniques as factors influencing the arithmetic mean roughness (Ra) [13] or have analyzed processes operating under dry conditions [14]. On the other hand, some of the authors have use other influencing parameters such as the chips’ characteristics [15] or tool geometry parameters [14,16,17,18,19].

In all the above-mentioned studies, the most often used workpiece materials were aluminum alloys, magnesium alloys, superalloys (Inconel 718 and Titanium alloy) and hardened steel.

The focus of this study is on the modeling of artificial neural networks for the prediction of the arithmetic mean roughness (Ra) in milling. Previous studies showed that neural networks can be applied for surface roughness predictions in different machining operations such as turning [5,9,10,11,14,16,19], milling [3,4,6,7,8,13,15,17,18,20,21,22] and drilling [12].

To reduce the cost of experiments, researchers often use the Taguchi design of experiments [5,6,15,17,20,21], although when the number of the factors and levels in the Taguchi design of the experiment is not too high, an experiment can be conducted as a full factorial [4,14]. The input-output datasets used in all the above-mentioned studies for handle surface roughness predictions can be considered small.

Many researchers have used the trial-and-error method to improve the performance of neural networks. That means the application of different network topologies, i.e., different numbers of hidden layers and numbers of neurons incorporated into them, different training algorithms, learning parameters, etc. Through this process, neural networks are tested and evaluated, and then the optimal structure is determined.

The neural networks most frequently used to predict the arithmetic mean roughness (Ra) are feed-forward backpropagation neural networks and radial basis functions. Munoz-Escalona et al. [15], Šarić et al. [13] and Fang et al. [16] compared both network types to find the optimal model, whereas Hossain et al. [6], Al Hazza et al. [3] and Zain et al. [18] used feed-forward backpropagation neural networks, etc.

Table 1 and Table 2 show a literature review of recent work on arithmetic mean roughness (Ra) prediction using neural networks. Table 1 contains data on the machining process, materials to cut and cutting conditions, and Table 2 contains information on the ANN type, the structure of the neural network and the dataset at disposal for network training.

Researchers have tested different network topologies and training algorithms to obtain good prediction results, and Benardos et al. [20] found that the 5-3-1 topology and the LM training algorithm showed the best performance. Similarly, Alharthi et al. [4] achieved good results in predicting Ra using a 3-6-1 neural network topology and the momentum algorithm. Al Hazza et al. [3] proposed a 3-20-4-4 network topology and an LM algorithm. Vardhan et al. [22] found that a network topology of 5-8-8-2 had the best prediction performance, etc.

From Table 2 it can be seen that dataset size varied from 18 up to 304 samples, but most of the presented studies used 27 samples. The ratio of the training, validation and testing samples varies depending on the training algorithm, so Al Hazza et al. [3] used a 70:15:15 ratio, Ezugwua et al. [10] 50:25:25 and 67:33, Zain et al. [18] 85:15, Alharthi et al. [4] 80:20, etc.

How big does a dataset need to be for one to make a performance prediction? The answer to this question is not simple and depends first of all on the problem’s complexity and the learning algorithm’s complexity. The way to check this is to train neural networks of different architectures using the available data set, perform a simulation and compare the results to the data that can be considered accurate.

In this paper, we present the development of artificial neural networks (ANNs) for the prediction of the arithmetic mean roughness, which is one of the most common surface roughness parameters. The dataset used for ANN development was obtained experimentally by machining AA7075 aluminum alloy under various machining conditions. The experiment was conducted based on the full factorial plan with four factors and three levels each, whereas the arithmetic mean roughness was selected as a response.

For four factors having three levels each, the full factorial design considers a total of 81 experimental runs that have to be carried out. Using input factor-level settings and adopting the Taguchi method, the experiments could be reduced from 81 runs to 27 runs by means of an orthogonal design.

The dataset used for ANN training contained 27 input-output parameters, corresponding to the L27 orthogonal array. A certain number of neural networks with different architectures was developed and evaluated.

The neural networks which showed the best performance after evaluation were then simulated. For network simulation, we used input pairs derived from the full factor plan, but these were not used in network training. The results of the ANN simulation were then compared with the output data set obtained experimentally.

Furthermore, this studied demonstrates the reliability of artificial neural networks based on a small input-output dataset.

## 2. Experimental Procedure

This paper investigates the effects of four factors, each having three levels, on the surface roughness: cutting speeds *v*, feed rate *f*, axial depth of cut *a* and the types of coolants/lubricants. Experimental factors and their levels are given in Table 3.

Experiments were conducted in a Emco Concept Mill 250 milling center, machining AA7075 aluminum alloy under various machining conditions. All experimental runs were performed under identical machining conditions and using the same machine tool. The chemical composition of AA7075 aluminum alloy is given in Table 4.

A solid carbide multi-flute end mill Φ12 × 33/80 series WAE303A was used for the experiment. This tool is designed for the cutting of aluminum alloys, brass and bronze, is uncoated and has three flutes, and the angle of the helix is 45 degrees.

The numbers of revolutions per minute used in the experiments (*n*_1_ = 3185 rpm, *n*_2_ = 4246 rpm, and *n*_3_ = 5308 rpm) were obtained based on adopted upper and lower cutting speed values, using the formula *n* = (1000 · *v*)/(*D · π*), where *D* is cutter diameter and *v* is cutting speed. The feed rate per minute is obtained based on the determined number of revolutions and selected feed per tooth (Table 3) using the formula *s = n∙s_z_∙z*, where *n* is rpm, *s_z_* is the feed per tooth and *z* is the cutting tool number of flutes. The minimum feed rate used in experiments (*s_min_* = 478 mm/min) corresponds to the smallest number of revolutions (*n*_1_ = 3185 rpm) and the lowest value of feed per tooth (*s_z_*_1_ = 0.05 mm/tooth). The maximum feed rate (*s_max_* = 2389 mm/min) corresponds to a maximum rpm (*n*_3_ = 5308 rpm) and maximum feed per tooth (*s_z_*_3_ = 0.15 mm/tooth).

**Figure 1:**
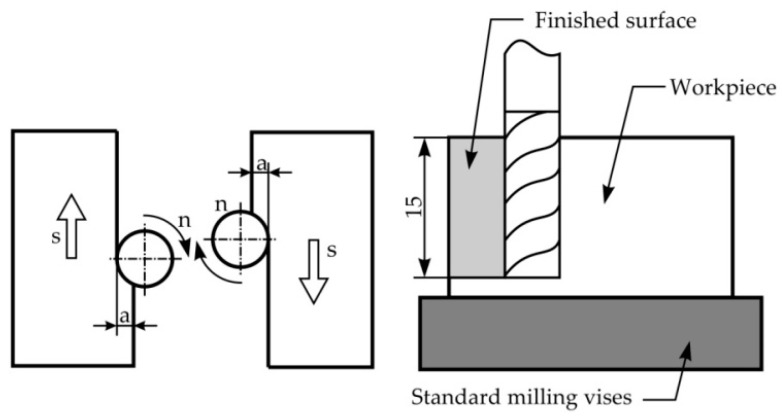
Down milling.

The sample parts were cut from a 20 mm-thick AA7075 plate to dimensions 30 × 30 × 20, fixed by a general-purpose milling vise. Machining was performed on both sample part sides using the climb milling method. In a milling operation, the sample parts were fed along the cutter’s direction of rotation, since this ensures that the machined surface has a better quality, Figure 1. The axial depth of cut was a1 = 0.6 mm, a2 = 0.8 mm and a3 = 0.1 mm, and the radial depth of the cut had a constant value of a = 15 mm. The following cooling/lubricating methods were used: air, dry cutting and emulsion.

The arithmetic mean roughness Ra was measured using a Mitutoyo SJ-210, Mitutoyo Europe GmbH, Neuss, Germany measuring device. The device had the following characteristics: cutoff filter values *λc* equal to 0.08, 0.25, 0.8 and 2.5 mm; a phase-matched Gaussian filter according to DIN EN ISO 16610-21; and measuring speeds when measuring of 0.25, 0.50 and 0.75 mm/s, when returning 1 mm/s. The measuring force/stylus type was 0.75 mN/2 μmR 60°. For this experiment, the settings of the device were determined according to the expected value of the Ra and were equal to *λf* = 2.5 μm, *λc* = 0.08 mm, *ln* = 4 mm.

Semi-synthetic metalworking fluid BIOL MIN-E, quality level ISO 6743/7. was used for wet machining. This fluid can be used for machining all types of steel and cast iron, non-ferrous and light metals.

## 3. Taguchi Method for Optimization of Cutting Parameters

The influence of various factors on the quality of the surface was determined experimentally. Implementation of the full factorial experiment means carrying out the maximum number of experimental runs determined by the number of factors and levels. This means a longer time and thus a higher cost of the experiment. The Taguchi method is one of the most commonly used methods in the design of experiments. This method allows fewer experimental runs compared to the full factorial plan. The main goal of this method is to define a minimum number of experimental runs, which will contain the optimal combination of factors and levels. Subsequent data analysis is based on statistical methods. It determines the optimal conditions, in order to obtain a minimum of the cost function. The Taguchi optimization method is often used to obtain low surface roughness in various cutting operations [23,24,25,26,27].

A measure of quality characteristics, the signal-to-noise ratio, is observed. The signal-to-noise ratio represents a deviation from the desired value.

For the optimization of static problems, there are three signal-to-noise ratios [28]:

- Smaller is better:

(1) $$\frac{S}{N}=-10log\left(\frac{1}{n}\overset{n}{\underset{i=1}{\sum }}y_{i}^{2}\right)\;$$

- Bigger is better:

(2) $$\frac{S}{N}=-10log\left(\frac{1}{n}\overset{n}{\underset{i=1}{\sum }}\frac{1}{y_{i}^{2}}\right)\;$$

- Nominal is the best:

(3) $$\frac{S}{N}=10log\frac{\bar{y}}{s_{y}^{2}}\;$$

In the above equations, *S/N* is the signal-to-noise ratio, *n* is the number of responses, *y_i_* is the response for the factor/level combination, $\bar{y}$ is the mean of the response for the factor/level combination and $s_{y}^{2}$ is the standard deviation of the response for a given factor/level combination. The categories of the *S/N* ratio are selected based on the characteristics of the quality.

The goal of this experiment was the determination of the arithmetical mean roughness (Ra), and the selected *S/N* ratio of interest corresponded to the “Smaller is better” criterion. In this case, a full factorial experiment entailed 3^4^ = 81 experimental runs. Using the Taguchi method, the experiments were reduced from 81 runs to 27 runs by means of an orthogonal design. The orthogonal matrix shown in Table 5 has 27 rows, representing the number of experimental runs.

The last column in Table 5 contains the *S/N* values based on measuring the obtained Ra values, using the “Smaller is better” criterion and taking into consideration Equation (1). Based on *S/N*, it is possible to find out which parameters are the most influential in regard to the arithmetical mean roughness value, Ra.

An arithmetic mean roughness (Ra) table for cutting speed, axial depth of cut, feed per tooth and cooling/lubricating techniques was created in an integrated manner and the results are presented in Table 4. A greater *S/N* value corresponds to better performance. Therefore, the optimal level of the arithmetic mean roughness (Ra) is the level with the greatest *S/N* value, obtained at cutting speed 120 m/min (level 1), axial depth of cut 0.8 mm (level 2), feed per tooth 0.05 mm/tooth (level 1) and air cooling (level 3).

**Figure 2:**
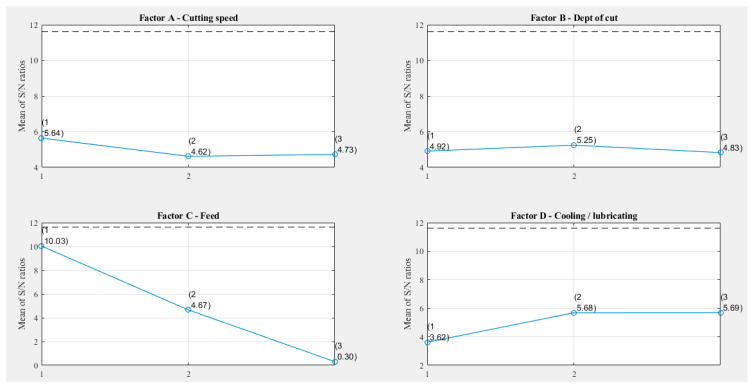
Main effect plot for *S/N* ratio.

Figure 2 shows *S/N* values for all factors/levels based on the data shown in Table 6. The slope of the line that joins the different parameter levels determines the contribution of each factor to the arithmetical mean roughness value, Ra. If the difference in the *S/N* ratio between levels is remarkable, the factor is more significant. The opposite is also true. If the difference in the *S/N* ratio from one level to another is negligible, that indicates that the factor is insignificant.

Figure 2 and Table 6 show that the feed rate had a more significant influence on the arithmetic mean roughness, Ra. The influence of the cooling and lubricating technique was less significant, whereas the influence of the depth of the cut and the cutting speed were the least significant. The optimization of the studied factors concerning the “Smaller is better” criterion provided the optimal combination, coded as 1-2-1-3. That implies the following input parameters: cutting speed 120 m/min, cutting depth 0.8 mm, feed per tooth 0.05 mm/tooth, and air cooling (Table 7).

The minimum of the arithmetical mean roughness, Ra, determined analytically based on the optimal input parameter combination, had a value of Ra = 0.262458. Since the 1-2-1-3 combination does not exist in the orthogonal array, a confirmation experiment needed to be conducted.

The experimentally obtained value of Ra for the 1-2-1-3 combination of input parameters was Ra = 0.2556 μm, which confirms the results obtained when applying the presented method.

## 4. ANOVA Technique

ANOVA is a statistical technique used to test the significance of the main factors comparing a mean square to an estimate of the experimental flaw at a certain level of confidence. In this study, the arithmetic mean roughness Ra obtained experimentally (Table 5, column 9) was analyzed using ANOVA. Analysis of variance illustrates the degree of importance of each factor that prominently influenced the arithmetic mean roughness Ra. Table 8 shows the ANOVA results for the arithmetic mean roughness Ra.

Columns 5 and 6 in Table 6 demonstrate the significance rates of the process parameters on the arithmetic mean roughness Ra, i.e., depicting whether the factors had a noticeable impact on the Ra.

Based on the F distribution tables, the ratios corresponding to 95% and 99% confidence levels are F _0.05, 2.18_ = 3.5560 and F _0.01, 2.18_ = 6.0129. That means that the cutting speed, feed rate and cooling/lubricating presented physical and statistical significance, since F > Fα = 5% and F > Fα = 1.

The most influential parameter affecting the surface roughness was the feed, at 91.392%. This also confirms results obtained using the Taguchi methodology, shown in Figure 2 and Table 6.

A relatively small error value of 1.254% indicates the following:

- The influence of parameters that we did not consider in the experiment on the arithmetic mean roughness, Ra, was negligible;
- The small value of the error indicates that the experiment can be considered successful.

## 5. Artificial Neural Networks

The artificial neural network is a data processing system inspired by the human biological neural network. ANNs use datasets obtained experimentally or analytically to model the behavior of a system with several influencing factors. An ANN has an input layer, one or more hidden layers and an output layer. Each layer consists of one or more elementary units called neurons [30]. The neuron is considered a processor, having one or more inputs and one output. A number of factors and the number of outputs define the number of neurons in the input and output layers. The number of neurons in the hidden layer is determined according to Equations (4)–(6) [31].

(4) $$n=\frac{2\left(N_{x}+N_{y}\right)}{3}$$

(5) $$n<2N_{x}$$

(6) $$n=\sqrt{N_{x}\cdot N_{y}}$$

A desirable approach in defining a neural network’s architecture is to create several different architectures (topologies) of artificial neural networks and apply different training algorithms, then performing an evaluation of the performance using different criteria. Of the multiple training methods for neural networks, this paper covers only supervised learning. Supervised learning involves providing the inputs and correct outputs, meaning that the network processes the inputs and compares the results to desired outputs. The errors (differences between desired outputs and network outputs) are backpropagated throughout the system, leading to adjustments of the weights which control the network. The dataset used for developing the ANNs contained 27 samples, belonging to the initial set of 81 samples. The NN model trained with 27 samples is then presented (in the simulation process) with an experimentally obtained input set. The simulation input set is completely unknown to the network since none of the elements belonging to it was used in training. The aim is to estimate the accuracy of the network trained using a small dataset. Matlab software, i.e., its graphical environment NNToolbox, was used for neural network development. Samples were first divided into three sets: 70% of the dataset (19 samples) was used for training, 15% of the dataset (four samples) was used for validation and 15% of the dataset (five samples) was used for testing.

The Matlab function dividend ensures that the network performs the same kind of data division when it is trained: the training dataset contained samples 1 to 19, the validation dataset contained samples 20 to 23, and the testing dataset contained samples 24 to 27. In total, 108 different neural models were developed, using 18 different network architectures. Each of the selected network architectures was trained using six learning algorithms: Levenberg–Marquardt (LM), Bayesian regulation (BR), resilient backpropagation (RP), gradient descent (GDX), quasi-Newton backpropagation (BFG) and scaled conjugate gradient backpropagation (SCG). The sigmoid and linear functions were used as transfer functions. The sigmoid transfer function was used between the input and hidden layer, sigmoid or linear were used between the hidden layers and the linear transfer function was used between the hidden and output layer.

Data Preprocessing

Several preprocessing techniques can be used to prepare numerical datasets. Linear scaling is one of them, in which variables are scaled linearly to adjust the weight ratios, usually in the interval from 0 to 1. In this paper, linear scaling of input and target variables was in the range 0.1–0.9 according to Equations (7) and (8) [9,10,32].

(7) $$x_{\mathrm{scal}}=\frac{x-x_{\min}}{x_{\max}-x_{\min}}\left({\overset{\text{¯}}{x}}_{\max}-{\overset{\text{¯}}{x}}_{\min}\right)$$

(8) $$y_{\mathrm{scal}}=\frac{y-y_{\min}}{y_{\max}-y_{\min}}\left({\overset{\text{¯}}{y}}_{\max}-{\overset{\text{¯}}{y}}_{\min}\right)$$

In this case, ${\overset{\text{¯}}{x}}_{\max}$ and ${\overset{\text{¯}}{y}}_{\max}$ represent the minimum or maximum value of the range in which the data are scaled, and x_max_, x_min_, y_max_ and y_min_ represent the largest and smallest values in the input and output data set, respectively. Experiments can be designed in many different ways.

Neural Network Evaluation

**Figure 3:**
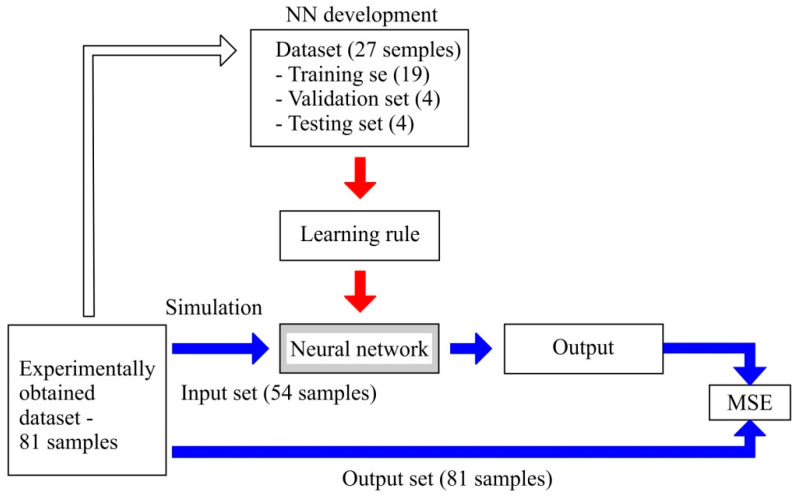
Simulation using the developed NNs and the experimentally obtained dataset [29].

Figure 3 shows the procedure of defining the input-output dataset based on the full factor plan, training the neural network with data corresponding to the L_27_ orthogonal array, and network simulation with an input data set. A total of 108 artificial neural networks with different architectures, as well as different training algorithms, were developed to predict the arithmetic mean roughness under different machining conditions (Appendix A). Out of that number, 18 networks with the best characteristics were selected. Estimation of characteristics was carried out based on the value of the mean square error (MSE) concerning the validation dataset and correlation coefficient (R) concerning the test dataset, Table 7. Next, each of the selected 18 models was simulated with input data set containing 81 samples derived from the experiment.

Of that number, 54 sets were completely unknown to the network, whereas 27 were known. The outputs from the simulation using the neural networks were then compared with the output set obtained experimentally. Based on the comparison of the absolute error of the simulation models output and the output determined experimentally, a conclusion was made about the qualityof the neural networks obtained based on a small set of data. Table 9 shows the mean square error between actual values obtained experimentally and estimated values (output of NN simulated by input set)

**Figure 4:**
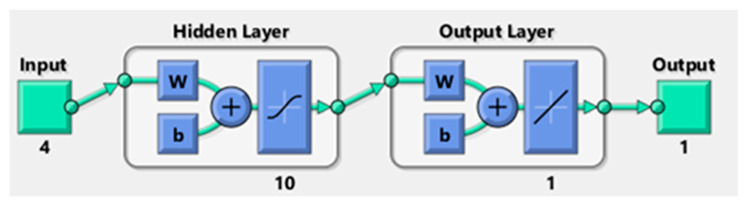
ANN architecture: 4 (10) 1.

The lowest value of the MSE 0.0025 had the neural network architecture of 4 (10) 1, trained using the Bayesian regulation (BR) algorithm (Figure 4).

## 6. Results and Discussions

**Figure 5:**
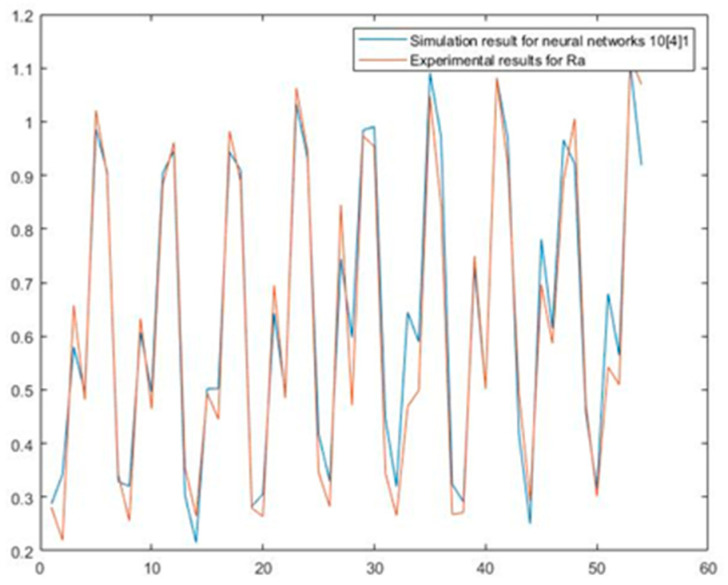
Predicted Ra values in comparison to test dataset.

After the neural network was selected, a simulation was carried out using the input/output dataset provided through the experiment. The input/output dataset used for simulation and to compare results contained 54 data, which were not used in the network training. Network performance was evaluated by comparing the results generated by the neural network and the experimentally determined output values. Figure 5 compares the arithmetic mean roughness (Ra) values predicted by the NN trained using the BR algorithm and Ra values obtained experimentally.

**Figure 6:**
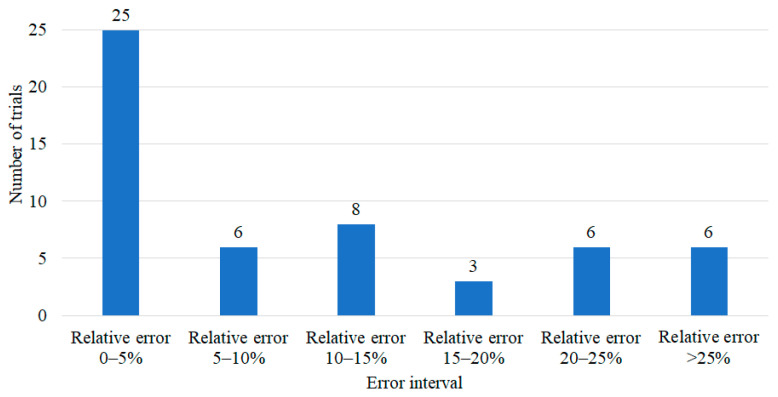
Neural network error distribution in arithmetic mean roughness prediction.

The relative error for 54 simulation outputs was calculated according to “Equation (9)”:

(9) $$\delta =\left|\frac{R_{aexp}-R_{aNN}}{R_{aexp}}\right|\times 100\%$$

where *δ* is the relative output error, *R_aexp_* is the value of the arithmetic mean roughness (Ra) determined experimentally and *R_aNN_* is the output value obtained by the network. Based on the relative error value, six error intervals were established (0–5%, 5–10%, 10–15%, 15–20%, 20–25% and more than 25%). Figure 6 shows the neural network error distribution in arithmetic mean roughness prediction.

**Figure 7:**
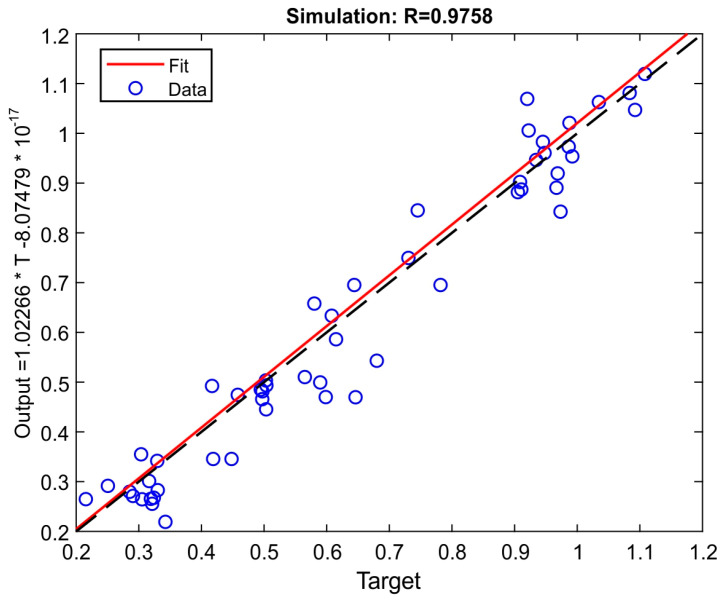
The plot of data regression (simulation).

The relatively high error value (above 20%) was due to the small range of output values. The regression plot for the simulation is shown in Figure 7, i.e., network outputs vs. experimentally-obtained outputs. If the network outputs were equal to the targets (experimentally obtained data), the data should fit a 45-degree line (dashed gray line). In this study, the fit can be considered good for all data sets, with R values of 0.9758 and linear regression coefficients x = 1.02266 and y = −8.07479 × 10^−17^ (red solid line). The mean square error (MSE) was equal to 0.00444271. The optimal combination of input parameters was 1-2-1-3. The minimum arithmetical mean roughness, Ra, was determined through the simulation using the neural network and this input set. The result after re-scaling was Ra = 0.32 μm.

## 7. Conclusions

The ability to predict surface roughness parameters under given machining conditions is important from an economic perspective as well as from the point of view of assessing the mechanical properties of a part and its function. This paper provides an overview of research related to the prediction of arithmetic mean roughness (Ra) using artificial neural networks in the end milling of aluminum alloy. The most frequently used factors in the presented studies were the feed rate, cutting speed and the depth of the cut. Moreover, different cooling/lubrication techniques, vibration and tool wear also have an impact on surface roughness.

In this paper, we analyzed the influence of the feed rate, cutting speed, radial depth of the cut and cooling/lubricating technique on surface roughness.

This study shows that:

1. When designing and conducting an experiment using the Taguchi technique, the number of samples is smaller in comparison to the full factor plan. In such a case, neural networks handle small datasets of experimental data. Bearing in mind that the set of available data is small, it is necessary to carefully plan the neural network topology and algorithms for training.
2. In this study, the dataset used for ANN development contained 27 samples. The full factorial plan was used to simulate and evaluate the neural network.
3. Improving neural network performance is possible through the trial-and-error method. This means including different training algorithms, different numbers of hidden layers and neurons, learning parameters, the transfer function, etc. Of the 108 developed neural network models, a topology consisting of four neurons in the input layer, one hidden layer with ten neurons and one neuron in the output layer (4-10-1) was found to have the lowest value of MSE, equal to 0.00444271. The 4-10-1 network structure was trained using the BR algorithm. The results and the levels of the mean square error (MSE) were acceptable in terms of the proposed model for the prediction of arithmetic mean roughness (Ra).
4. Twenty-seven input-output pairs were consider as a small dataset in the context of neural network modeling. This study shows that it is possible to obtain a good prediction and that the small dataset is not an obstacle.
5. Compared to conventional methods, the advantages of using ANNs are their higher speed, simplicity and the possibility of learning based on examples.
6. A disadvantage is that the application of this method requires an experimentally determined dataset, which can be expensive and time-consuming in some cases.

The presented research shows that artificial neural networks can be used to predict arithmetic mean roughness (Ra) in a short time and with good reliability, despite using a small dataset. Tackling the reverse problem can be a way of extending this research. That approach would imply defining the cutting parameters based on the desired value of surface roughness.

## Figures and Tables

**Table 1:** ANN for prediction of the arithmetic mean roughness (Ra) in different machining processes.

Author, Year	Work Material	Process	Cutting Conditions
Munoz-Escalona, et al. (2010) [15]	AA7075-T7351	Face milling	Experimental input parameters: Cutting speed (600, 800, 1000 m/min), feed rate (0.1, 0.2, 0.3 mm/tooth), axial depth of cut (3, 3.5, 4 mm). Feature extraction: chip width, and chip thickness.
Benardos et al. (2002) [20]	AA Series 2	Face milling	Experimental input parameters: Cutting speed (300, 500, 700 m/min), feed rate (0.08, 0.14, 0.2 mm/tooth), depth of cut (0.25, 0.75, 1.2 mm), tool engagement (30%, 60%, 100%), cutting fluid (yes, no).
Alharthi et al. (2017) [4]	AZ61 magnesium alloy	Face milling	Experimental input parameters: Cutting speed (500, 1000, 1500, 2000 m/min), feed rate (50, 100, 150, 200 mm/min), depth of cut (0.5, 1, 1.5, 2 mm).
Šarić et al. (2013) [13]	S235JRG2 steel	Face milling	Experimental input parameters: Number of revolutions (400, 600, 800 rpm), feed rate (100, 300, 500 mm/min), depth of cut (0.5, 1, 1.5 mm), cooling /lubricating technique: without cooling, through the tool, outside cooling.
Kadirgamaa et al. (2008) [8]	AA6061-T6	End milling	Experimental input parameters: Cutting speed (100, 140, 180 m/min), feed rate (0.1, 0.15, 0.2 mm/rev), axial depth of cut (0.1, 0.15, 0.2 mm), radial depth of cut (2, 3.5, 5 mm).
Huang et al. (2008) [7]	AA6061	End milling	Experimental input parameters: Cutting speed (1750, 1800, 1850, 1900, 2050, 2100, 2200, 2250, 2300, 2400, 2500 rpm) depth of cut (0.04, 0.05, 0.06, 0.07, 0.08) and feed rate (6, 8, 10, 12, 13, 14, 15, 16, 17, 18, 19, 20/min).
Karagiannis et al. (2014) [17]	AA5083	End milling	Experimental input parameters: Cutting speed (5000, 6000, 7000 rpm), depth of cut (0.5, 1, 1.5 mm), and feed rate (0.05, 0.08, 0.1 mm/tooth) Tool geometry parameters: core diameter (%), flute angle (°), rake angle (°), first relief angle (°) and second relief angle (°). Output parameters: Ra, Ry, Rz.
Hossain et al. (2008) [6]	Inconel 718	End milling	Experimental input parameters: Cutting speed (20, 30, 40 m/min), axial depth of cut (0.4, 0.6, 0.8 mm) and feed rate (0.04, 0.075, 0.11 mm/tooth).
Al Hazza et al. (2013) [3]	AISI H13	End milling	Experimental input parameters: Cutting speed 150 up to 250 m/min, feed rate 0.05–0.15 mm/rev and depth of cut 0.1–0.2 mm. Output parameters Ra, Rt, Rz, Rq.
Zain et al. (2010) [18]	Titanium alloy (Ti-6A1-4V)	End milling	Experimental input parameters: Cutting speed (124.53, 130, 144.22, 160, 167.03 m/min), feed rate (0.025, 0.03, 0.046, 0.07, 0.083 mm/tooth), radial rake angle (6.2, 7, 9.5, 13.0, 14.8°).
Vardhan et al. (2018) [22]	P20	Milling	Experimental input parameters: Nose radius (0.8, 1.2 mm), cutting speed (75, 80, 85, 90, 95 m/min), feed rate (0.1, 0.125, 0.75, 1, 1.25, 1.5 mm/tooth), axial depth of cut (0.5, 0.5, 0.8 mm), radial depth of cut (0.3, 0.4, 0.5, 0.6, 0.7 mm). Output parameters: material removal rate (MRR) and surface roughness (Ra).
Eser et al. (2021) [21]	AA6061	Milling	Experimental input parameters: Cutting speed (100, 150, 200 m/min), depth of cut (1, 1.5, 2 mm) and feed rate (0.1, 0.15, 0.2 mm/rev)
Fang et al. (2016) [16]	AA 2024-T351	Turning	Experimental input parameters: Cutting speed (150, 250, 350 m/min), depth of cut (0.8 mm constant), tool nose radius (0.8 mm constant), feed rate (varied at five levels based on the ratio to the tool radius: 1.0, 1.5, 2.0, 2.5, and 3.0).
Krishnan et al. (2019) [11]	AA6063	Turning	Experimental input parameters: Cutting speed (2000 rpm), feed rate (0.1 mm/rev), depth of cut (0.5 mm). Feature extraction: frequency range grayscale value, major peak frequency (F1), and the principal component magnitude squared (F2).
Kumar et al. (2015) [14]	AA7075/10/SiCp and AA7075 Hybrid Composites	Turning	Experimental input parameters: Cutting speed (80, 110, 140, 170 m/min), feed rate (0.05, 0.1, 0.15, 0.2 mm/rev), approaching angle (45, 60, 75, 90°). Experiments were conducted without cooling/lubricating media (dry cutting).
Zhong et al. (2006) [19]	Aluminum and copper	Turning	Experimental input parameters: Tool insert grade (TiAlN coated carbide, PCD), tool insert nose radius (0.2, 0.4, 0.8 mm), tool insert rake angle (0, +5, +15 deg), work piece material (aluminum, coper), cutting speed (500, 1000, 2500 rev/min), feed rate (0.01, 0.1, 0.2 rev/min), depth of cut (0.05, 0.5, 1 mm). Output parameters: Surface roughness parameters R_a_, R_t_.
Pal et al. (2005) [9]	Mild steel	Turning	Experimental input parameters: Cutting speed (325, 420, 550 m/min), depth of cut (0.2, 0.5, 0.8 mm) and feed rate (0.04, 0.1, 0.2 mm/rev).
Beatrice et al. (2014) [5]	Hardened steel H13	Turning	Experimental input parameters: Feed rate (0.05, 0.075, 0.1 mm/rev), cutting speed (75, 95, 115 m/min), depth of cut (0.5, 0.75, 1 mm).
Ezugwu et al. (2005) [10]	Inconel 718	Turning	Experimental input parameters: Cutting speed (20, 30, 40, 50 m/min), feed rate (0.25, 0.30 mm/rev), cutting time (312, 774 s) and the coolant delivery pressure (110, 150, 203 bar). Seven output parameters were observed: cutting force (Fz) and feed force (Fx), power consumption (P), surface roughness (Ra), average flank wear (VB), maximum flank wear (VBmax), nose wear (VC).
Vrabel et al. (2012) [12]	Udimet 720	Drilling	Experimental input parameters: Feed rate, cutting speed, thrust force. Output parameters: drill flank wear VB and surface roughness.

**Table 2:** ANN types, network structure, and dataset for network training.

Author, Year	ANN Types, Training Algorithm, Network Topology	Dataset	Remarks
Munoz-Escalona, et al. (2010) [15]	The radial basis function NN (RBF NN), generalized regression (GRNN) networks, and feed forward back propagation neural network (FFBP NN) were compared.	Taguchi design of DoE, L9 orthogonal array.	The presented results show a good correlation between the surface roughness and thickness of the chip. FFBP neural network shows better results than radial basis network.
Benardos et al. (2002) [20]	FFBP NN Training algorithm: Levenberg–Marquardt (LM) Network topology 5-3-1 showed the best performance.	The dataset contained 27 samples (18 used for training, 4 for validation, 5 for testing).	ANN was able to predict the surface roughness with a mean squared error (MSE) equal to 1.86%.
Alharthi et al. (2017) [4]	FFBP NN Training algorithms: LM, Momentum Network topology 3-6-1 showed the best performance.	The experiment was conducted based on a full factorial plan. The dataset contained 64 samples (80% training set, 20% testing, and validation set).	This paper analyzed two models: ANN and regression model. The predicted values of Ra were compared to the results of the experiment. Both models could predict Ra with high accuracy. The determination coefficients were 95% for the best neural network and 94% for regression analysis.
Šarić et al. (2013) [13]	Several different NN models were analyzed: RBF NN, FFBP NN, modular NN (MNN) Training algorithm—not stated. Optimal network topology—not stated.	Not stated.	Different learning rules and transfer functions were analyzed. For all three NN types the best results were obtained with the sigmoid transfer function. The Delta learning rule provided the best results for FFBP NN and MNN, whereas the normalized-cumulative delta provided the best results for RBF NN. Results show that all networks could be implemented and efficiently in surface roughness prediction.
Kadirgamaa et al. (2008) [8]	RBF NN compared to response surface method (RSM).	Not stated.	RBF NN can predict the arithmetic mean roughness more accurately than RSM.
Huang et al. (2008) [7]	FFBP NN, Training algorithm—not stated. Network topology 5-8-7-1 showed the best performance.	The dataset contained 336 samples.	After launching the adaptive control function, the arithmetic mean roughness Ra value was smaller.
Karagiannis et al. (2014) [17]	FFBP NN Training algorithm—not stated. Network topology 8-7-5-4-3 showed the best performance.	The dataset contained 18 samples based on an L18 (2^1^ × 3^7^) orthogonal array.	The network had 3 output parameters: Ra, Ry, Rz. Coefficient of correlation during training R = 1, validation R = 0.89, testing R = 0.93. For enhancement of the FFBP NN model, researchers needed to increase the number of trials.
Hossain et al. (2008) [6]	FFBP NN Training algorithm LM Network topology—not stated.	The dataset contained 27 samples.	NN had very good predictive performance.
Al Hazza et al. (2013) [3]	FFBP NN Training algorithm LM Network topology 3-20-4-4 showed the best performance.	The dataset contained 20 samples, a data ratio of 70:15:15.	Predicted and experimental data were in good agreement.
Zain et al. (2010) [18]	FFBP NN Training algorithm: trained (gradient descent with momentum and adaptive learning rule BP). Network topology 3-1-1 showed the best performance.	The dataset contained 24 samples, with a data ratio of 85:15.	NN model could predict Ra using a small dataset. The small number of samples was not a hindrance to obtaining good prediction results.
Vardhan et al. (2018) [22]	FFBP NN Training algorithm—not stated. Network topology 5-8-8-2 showed the best performance.	The dataset contained 50 samples based on L50 (2^1^ × 5^4^) orthogonal array.	The developed ANN network predicted the arithmetic mean roughness Ra and MRR with a deviation of 4.3785% and 17.45823% compared to the test data set.
Eser et al. (2021) [21]	FFBP NN Five training algorithms: Broyden–Fletcher–Goldfarb–Shanno (BFGS), central pattern generator (CPG), Levenberg–Marquardt (LM), resilient backpropagation (RP), scaled conjugate gradient (SCG) Network topology—not stated.	The dataset contained 27 samples.	The results of comparing RSM and ANN models were presented in this research. Both models provided results very close to experimentally obtained ones. The ANN trained using the SCG algorithm showed the best results.
Fang et al. (2016) [16]	Two NN models were used: FFBP NN and RBF NN. Training algorithm and network topology—not stated.	The dataset contained 45 samples (38 training sets, 7 test sets).	This paper presented a comparison of the prediction of the arithmetic mean roughness (Ra) obtained using the RBF and MLP models. The second model provided better results, especially in the prediction of maximum roughness height.
Krishnan et al. (2019) [11]	FFBP NN Training algorithm—not stated. Network topology 6-10-1 showed the best performance.	The dataset contained 40 samples.	The shown methodology used an ANN to detect the errors in the surface roughness of the materials.
Kumar et al. (2015) [14]	FFBP NN Training algorithm—variable learning rate backpropagation (GDX) The neural network had 2 hidden layers and 10 neurons.	The experiment was conducted based on a full factorial plan. The dataset contained 64 samples.	Response surface methodology (RSM) was used for the analysis of the experimental data. ANN prediction and RSM were compared to experimental data. The correlation coefficient of the RMS to the experiment was 0.9972 and that of the ANN to the experiment 0.99571. Results showed that the ANN had a greater deviation than the RSM prediction.
Zhong et al. (2006) [19]	FFBP NN Training algorithm—not stated. Network topology 7-14-18-2 showed the best performance	The dataset contained 304 samples (274 training sets, 30 testing sets).	Surface roughness parameters predicted by the neural network were in good agreement with experimentally obtained ones.
Pal et al. (2005) [9]	FFBP NN, Training algorithm—not stated. NN 5-5-1 showed the best performance	The dataset had 27 samples; 20 were used for training and 7 for testing.	Predicted surface roughness was compared with experimental data and was in good agreement.
Beatrice et al. (2014) [5]	FFBP NN Training algorithm LM. Network topology 3-7-7-1 showed the best performance in surface roughness prediction.	The dataset contained 27 samples based on the L_27_ orthogonal array. Out of that number 23 samples were used for training and 4 for testing.	A neural network model was developed using a small dataset. Despite this, the model predicted the arithmetic mean roughness with considerable accuracy, since the error between the NN model simulation results and experimentally obtained ones was less than 7%.
Ezugwu et al. (2005) [10]	FFBP NN Training algorithms: LM and Bayesian regularization (BR). Network topology 5-10-10-7 trained by the BR algorithm showed the best performance.	The dataset contained 102 samples. LM algorithm dataset ratio: 50:25:25 BR algorithm dataset ratio 67:33.	Two neural networks were made, having one and two layers, 10 and 15 neurons in each, trained by two algorithms, LM and BR. The best results were shown by a neural network trained using the BR algorithm, with two layers and 15 neurons in the hidden layer.
Vrabel et al. (2012) [12]	FFBP NN Training algorithm—not stated. NN 3-4-1 and 3-5-1 showed the best performance in tool wear prediction and NN 4-6-1 and 4-6-4-1 in surface roughness prediction.	Dataset had 42 samples; 32 were used for training and 10 for testing.	NN 3-5-1 was used in the prediction of tool wear. The average RMS error was 12.7%. NN 4-6-4-1 was used for the prediction of surface roughness, with an average RMS error of 2.64%.

**Table 3:** Experimental factors and their levels.

Factor	Level		
	1	2	3
Cutting speed (m/min)	120	160	200
Axial dept of cut (mm)	0.6	0.8	1
Feed per tooth (mm/tooth)	0.05	0.1	0.15
Cooling/lubricating technique	Emulsion	Dry cut	Air

**Table 4:** The chemical composition of AL 7075 aluminum alloy.

Element	Fe	Si	Cu	Mn	Mg	Zn	Cr	Ti	Other Each	Al
%	0.5	0.4	1.2–2	0.3	2.1–2.9	5.1–6.1	0.21	0.21	0.15	Balance

**Table 5:** Experimental plan—L_27_ orthogonal array (Taguchi method) [29].

Number of Trials	Cutting Speed (m/min)	Axial Depth of Cut (mm)	Feed Per Tooth (mm/tooth)	Cooling/Lubricating Techniques	Replicate			Average Ra (µm)	*S/N* Ratio (dB)
					Ra 1 (µm)	Ra 2 (µm)	Ra 3 (µm)		
1	120	0.6	0.05	Emulsion	0.348	0.361	0.345	0.3513	9.085613
2	120	0.6	0.1	Dry machining	0.477	0.476	0.476	0.476	6.441781
3	120	0.6	0.15	Air cooling	0.936	0.925	0.926	0.929	0.639686
4	120	0.8	0.05	Dry machining	0.264	0.262	0.259	0.261	11.68051
5	120	0.8	0.1	Air cooling	0.515	0.514	0.516	0.515	5.763855
6	120	0.8	0.15	Emulsion	0.973	0.971	0.973	0.972	0.243697
7	120	1	0.05	Air cooling	0.261	0.259	0.259	0.260	11.71168
8	120	1	0.1	Emulsion	0.574	0.689	0.633	0.632	3.98291
9	120	1	0.15	Dry machining	0.868	0.865	0.87	0.868	1.232942
10	160	0.6	0.05	Emulsion	0.327	0.39	0.40	0.369	8.665359
11	160	0.6	0.1	Dry machining	0.529	0.499	0.501	0.510	5.854275
12	160	0.6	0.15	Air cooling	0.939	0.929	0.935	0.934	0.589963
13	160	0.8	0.05	Dry machining	0.319	0.319	0.315	0.318	9.960567
14	160	0.8	0.1	Air cooling	0.566	0.569	0.563	0.566	4.943671
15	160	0.8	0.15	Emulsion	1.082	1.088	1.083	1.084	−0.70326
16	160	1	0.05	Air cooling	0.309	0.301	0.306	0.305	10.30452
17	160	1	0.1	Emulsion	0.825	0.809	0.809	0.814	1.783956
18	160	1	0.15	Dry machining	0.978	0.973	0.975	0.975	0.216939
19	200	0.6	0.05	Emulsion	0.434	0.419	0.413	0.422	7.493751
20	200	0.6	0.1	Dry machining	0.558	0.598	0.575	0.577	4.776484
21	200	0.6	0.15	Air cooling	0.923	0.93	0.919	0.924	0.686561
22	200	0.8	0.05	Dry machining	0.3	0.306	0.296	0.300	10.43829
23	200	0.8	0.1	Air cooling	0.53	0.514	0.519	0.521	5.663246
24	200	0.8	0.15	Emulsion	1.088	1.095	1.088	1.090	−0.75119
25	200	1	0.05	Air cooling	0.284	0.282	0.287	0.284	10.92344
26	200	1	0.1	Emulsion	0.723	0.732	0.725	0.727	2.773295
27	200	1	0.15	Dry machining	0.938	0.938	0.943	0.940	0.540524

**Table 6:** *S/N* ratio of the arithmetic mean roughness (Ra).

Factors	Level			Delta	Rank
	1	2	3		
Cutting speed	5.64	4.62	4.73	1.02	3
Axial dept of cut	4.92	5.25 *	4.83	0.42	4
Feed per tooth	10.03 *	4.67	0.30	9.73	1
Cooling/lubricating techniques	3.62	5.68	5.69 *	2.07	2

* Optimal level.

**Table 7:** Optimum factor levels.

	Speed	Dept of Cut	Feed	Cooling/Lubricating	*S/N*	Ra Calculated	Ra Test
Level	1	2	1	3	11.619	0.262458	0.2556

**Table 8:** Results of ANOVA for the arithmetic mean roughness.

Factor	DOF	Sum of Squares	Variance	F	Percent
A	2	0.024	0.012	8.347	1.163%
B	2	0.005	0.957	1.884	0.263%
C	2	1.914	0.003	659.569	91.932%
D	2	0.112	0.056	38.654	5.388%
Error	18	0.03	0.001		1.254%
Total	26	2.08			100%

**Table 9:** MSE between actual values (output set obtained experimentally) and estimated values (output of NN simulated by input set).

No.	Training Algorithm	ANN Architecture	MSE
1	SCG	4 (1) 1	0.0053
2	SCG	4 (3) 1	0.0305
3	SCG	4 (4) 1	0.00997
4	SCG	4 (2-2) 1	0.0225
5	SCG	4 (5-2) 1	0.0063
6	SCG	4 (5-2-3) 1	0.0102
7	BFG	4 (1) 1	0.0127
8	BFG	4 (10-4) 1	0.0172
9	BFG	4 (10-4-2) 1	0.015
10	GDX	4 (1) 1	0.0043
11	GDX	4 (3) 1	0.0194
12	GDX	4 (8) 1	0.0680
13	RP	4 (1) 1	0.00348
14	RP	4 (8-4) 1	0.01593
15	BR	4 (10) 1	0.0025
16	BR	4 (1-1) 1	0.0028
17	LM	4 (5-2) 1	0.00823
18	LM	4 (10-4-2) 1	0.01287

## Data Availability

The data that support the findings of this study are available from the corresponding author, upon reasonable request.

## Appendix A

**Table A1:** Results for All NN Models [29].

	NN Architecture	R	Number of Epochs	MSE		NN Architecture	R	Number of Epochs	MSE
	Levenberg–Marquardt (LM) algorithm					Bayesian regulation (BR) algorithm			
1	4 × 1 × 1	0.91322	6	0.0020	1	4 × 1 × 1	0.98901	41	0.00189
2	4 × 2 × 1	0.9556	11	0.0019	2	4 × 2 × 1	0.99467	44	0.00096
3	4 × 3 × 1	0.97698	6	0.0027	3	4 × 3 × 1	0.99430	97	0.00027
4	4 × 4 × 1	0.983	9	0.0047	4	4 × 4 × 1	−0.9837	58	0.056499
5	4 × 8 × 1	0.979	7	0.006	5	4 × 8 × 1	0.98222	125	2.14 × 10^−14^
6	4 × 10 × 1	0.799	6	0.014	6	4 × 10 × 1	0.98051	173	1.75 × 10^−14^
7	4 × 1 × 1 × 1	0.976	7	0.0012	7	4 × 1 × 1 × 1	0.98909	1000	0.00189
8	4 × 2 × 2 × 1	0.975	8	0.0057	8	4 × 2 × 2 × 1	0.98356	37	0.0577
9	4 × 3 × 2 × 1	0.943	6	0.0012	9	4 × 3 × 2 × 1	0.9838	37	0.0577
10	4 × 5 × 2 × 1	0.9964	9	0.00074	10	4 × 5 × 2 × 1	0.9838	38	0.0577
11	4 × 8 × 4 × 1	0.846	8	0.027	11	4 × 8 × 4 × 1	0.98402	38	0.0577
12	4 × 10 × 4 × 1	0.890	8	0.019	12	4 × 10 × 4 × 1	0.9838	38	0.0577
13	4 × 2 × 2 × 2 × 1	0.960	19	0.032	13	4 × 2 × 2 × 2 × 1	−0.78219	86	0.0577
14	4 × 3 × 2 × 2 × 1	0.965	17	0.0018	14	4 × 3 × 2 × 2 × 1	0	21	0.0667
15	4 × 4 × 3 × 2 × 1	0.9837	15	0.0013	15	4 × 4 × 3 × 2 × 1	0	35	0.0643
16	4 × 5 × 3 × 2 × 1	0.960	8	0.013	16	4 × 5 × 3 × 2 × 1	0.91736	32	0.0662
17	4 × 7 × 4 × 2 × 1	0.950	12	0.006	17	4 × 7 × 4 × 2 × 1	0	31	0.0664
18	4 × 10 × 4 × 2 × 1	0.99713	4	0.00020	18	4 × 10 × 4 × 2 × 1	0.90476	41	0.0658
	Resilient backpropagation (RP) algorithm					Gradient descent (GD ×) algorithm			
1	4 × 1 × 1	0.97513	6	3.16 × 10^−5^	1	4 × 1 × 1	0.97565	141	0.00058136
2	4 × 2 × 1	0.9436	6	0.010833	2	4 × 2 × 1	−0.9199	56	0.006586
3	4 × 3 × 1	0.9633	6	0.0024626	3	4 × 3 × 1	0.94019	140	0.0042864
4	4 × 4 × 1	0.82739	6	0.0044551	4	4 × 4 × 1	0.5879	88	0.0028835
5	4 × 8 × 1	0.97874	6	0.012403	5	4 × 8 × 1	0.7549	186	0.0060558
6	4 × 10 × 1	0.99259	11	0.021152	6	4 × 10 × 1	−0.91372	6	0.031521
7	4 × 1 × 1 × 1	0.9148907	16	0.0011574	7	4 × 1 × 1 × 1	0.82356	74	0.0037192
8	4 × 2 × 2 × 1	0.29668	7	0.019677	8	4 × 2 × 2 × 1	0.27105	81	0.033783
9	4 × 3 × 2 × 1	0.053017	8	0.039857	9	4 × 3 × 2 × 1	0.89452	77	0.042865
10	4 × 5 × 2 × 1	0.95375	17	0.010477	10	4 × 5 × 2 × 1	0.91328	117	0.012526
11	4 × 8 × 4 × 1	0.99162	19	0.012794	11	4 × 8 × 4 × 1	0.76334	74	0.0075099
12	4 × 10 × 4 × 1	0.96849	6	0.0019671	12	4 × 10 × 4 × 1	0.30189	54	0.020986
13	4 × 2 × 2 × 2 × 1	0.96881	30	0.0028199	13	4 × 2 × 2 × 2 × 1	−0.903229	77	0.03963
14	4 × 3 × 2 × 2 × 1	−0.055383	9	0.019247	14	4 × 3 × 2 × 2 × 1	−0.24808	77	0.03962
15	4 × 4 × 3 × 2 × 1	0.40091	10	0.034651	15	4 × 4 × 3 × 2 × 1	0.92829	116	0.023847
16	4 × 5 × 3 × 2 × 1	0.96214	18	0.0026711	16	4 × 5 × 3 × 2 × 1	0.93969	64	0.037863
17	4 × 7 × 4 × 2 × 1	0.93932	15	0.002873	17	4 × 7 × 4 × 2 × 1	0.9381	6	0.034819
18	4 × 10 × 4 × 2 × 1	0.93649	15	0.0023631	18	4 × 10 × 4 × 2 × 1	0.95615	124	0.025836
	Quasi-Newton backpropagation (BFG) algorithm					Scaled conjugate gradient backpropagation (SCG) algorithm			
1	4 × 1 × 1	0.97722	13	0.002691	1	4 × 1 × 1	0.97859	15	0.0017497
2	4 × 2 × 1	0.92073	32	0.0044498	2	4 × 2 × 1	0.92591	17	0.012217
3	4 × 3 × 1	0.9232	16	0.007515	3	4 × 3 × 1	0.88958	12	0.0026530
4	4 × 4 × 1	0.97149	14	0.0024259	4	4 × 4 × 1	0.96801	15	0.00099602
5	4 × 8 × 1	0.78725	25	0.0044701	5	4 × 8 × 1	0.77885	24	0.0061669
6	4 × 10 × 1	0.89511	42	0.0075095	6	4 × 10 × 1	0.9683	75	0.0030131
7	4 × 1 × 1 × 1	0.9739	16	0.0023739	7	4 × 1 × 1 × 1	0.98276	21	0.0005
8	4 × 2 × 2 × 1	0.94194	45	0.0025083	8	4 × 2 × 2 × 1	0.9474	13	0.0070227
9	4 × 3 × 2 × 1	0.89233	7	0.042685	9	4 × 3 × 2 × 1	−0.727	7	0.04
10	4 × 5 × 2 × 1	0.96475	27	0.004755	10	4 × 5 × 2 × 1	0.97979	27	0.0015945
11	4 × 8 × 4 × 1	0.93033	18	0.022565	11	4 × 8 × 4 × 1	0.91423	14	0.01604
12	4 × 10 × 4 × 1	0.90857	14	0.00099236	12	4 × 10 × 4 × 1	0.81501	6	0.04416
13	4 × 2 × 2 × 2 × 1	0.84936	13	0.026458	13	4 × 2 × 2 × 2 × 1	0.055	12	0.047
14	4 × 3 × 2 × 2 × 1	0.96006	14	0.013682	14	4 × 3 × 2 × 2 × 1	−0.0726	7	0.040627
15	4 × 4 × 3 × 2 × 1	0.057505	12	0.040046	15	4 × 4 × 3 × 2 × 1	0.899	11	0.0083887
16	4 × 5 × 3 × 2 × 1	0.90791	9	0.017959	16	4 × 5 × 3 × 2 × 1	0.97423	28	0.0096273
17	4 × 7 × 4 × 2 × 1	0.78003	9	0.032067	17	4 × 7 × 4 × 2 × 1	0.463	10	0.025448
18	4 × 10 × 4 × 2 × 1	0.96165	20	0.0037442	18	4 × 10 × 4 × 2 × 1	0.67	14	0.014792
